# Creating European guidelines for Chiropractic Incident Reporting and Learning Systems (CIRLS): relevance and structure

**DOI:** 10.1186/2045-709X-19-9

**Published:** 2011-04-01

**Authors:** Martin Wangler, Ricardo Fujikawa, Lise Hestbæk, Tom Michielsen, Timothy J Raven, Haymo W Thiel, Beatrice Zaugg

**Affiliations:** 1 European Academy of Chiropractic, academic dean and private practice, Bahnhofstrasse 15, 3400 Burgdorf, Switzerland; 2 Real Centro Universitario Maria Cristina-College of Chiropractic, Paseo de los Alamillos 2, 28200 San Lorenzo de El Escorial, Spain; 3 Nordisk Institut for Kiropraktik og Klinisk Biomekanik Forskerparken 10, 5230 Odense M, Denmark; 4 European Academy of Chiropractic, Department of Research and private practice, St. Paulusstraat 15, 2400 Mol-Belgium; 5 European Council on Chiropractic Education and private practice, Kiropraktorklinikken Strandgården, Havnegt. 1, 4790 Lillesand, Norway; 6 Anglo-European College of Chiropractic, 13-15 Parkwood Road, Bournemouth BH5 2DF, UK; 7 Swiss Academy of Chiropractic and private practice, Johann-Verresius-Str. 18 2502 Biel, Switzerland

## Abstract

**Background:**

In 2009, the heads of the Executive Council of the European Chiropractors' Union (ECU) and the European Academy of Chiropractic (EAC) involved in the European Committee for Standardization (CEN) process for the chiropractic profession, set out to establish European guidelines for the reporting of adverse reactions to chiropractic treatment. There were a number of reasons for this: first, to improve the overall quality of patient care by aiming to reduce the application of potentially harmful interventions and to facilitate the treatment of patients within the context of achieving maximum benefit with a minimum risk of harm; second, to inform the training objectives for the Graduate Education and Continuing Professional Development programmes of all 19 ECU member nations, regarding knowledge and skills to be acquired for maximising patient safety; and third, to develop a guideline on patient safety incident reporting as it is likely to be part of future CEN standards for ECU member nations.

**Objective:**

To introduce patient safety incident reporting within the context of chiropractic practice in Europe and to help individual countries and their national professional associations to develop or improve reporting and learning systems.

**Discussion:**

Providing health care of any kind, including the provision of chiropractic treatment, can be a complex and, at times, a risky activity. Safety in healthcare cannot be guaranteed, it can only be improved. One of the most important aspects of any learning and reporting system lies in the appropriate use of the data and information it gathers. Reporting should not just be seen as a vehicle for obtaining information on patient safety issues, but also be utilised as a tool to facilitate learning, advance quality improvement and to ultimately minimise the rate of the occurrence of errors linked to patient care.

**Conclusions:**

Before a reporting and learning system can be established it has to be clear what the objectives of the system are, what resources will be required and whether the implementing organisation has the capacity to operate the system to its full advantage. Responding to adverse event reports requires the availability of experts to analyse the incidents and to provide feedback in a timely fashion. A comprehensive strategy for national implementation must be in place including, but not limited to, presentations at national meetings, the provision of written information to all practitioners and the running of workshops, so that all stakeholders fully understand the purposes of adverse event reporting. Unless this is achieved, any system runs the risk of failure, or at the very least, limited usefulness.

## Introduction

Incident reporting is not new. Flannagan, a psychologist, first described the "critical incident technique" in 1954[1]. The concept originated from studies in the Aviation Psychology Program of the United States Air Force during and after the Second World War, with the aim of reducing the number of deaths of military pilots and the loss of aircraft[2,3].

The first health profession to use a critical incident technique was dentistry[4]. In 1960 the concept was introduced into medicine to identify errors in the dispensing of drugs by nurses[5]. By the 1970s the use of incident reporting became more widespread within the medical profession and was then employed as a mechanism to assess patient care given by medical specialists such as paediatricians, surgeons and obstetricians[6]. But it was not until 1978 that critical incident reporting was applied with the specific aim to improve patient safety. In the field of anaesthesia, Cooper and his colleagues[7] were the first to proactively put the emphasis of their investigations on "what could go wrong", rather than just analysing "what did go wrong" after an event had occurred. Anaesthesiology has since been regarded as the pioneer of critical incident studies in medicine. The purpose of any critical incident reporting system can be summarised as follows:

a. The occurrence of an incident should trigger the completion of a report;

b. Incident report data should then be collected and;

c. Analysed centrally to determine whether there are any trends that could represent potential problems in the delivery of care;

d. The results of the analysis must then be distributed and shared with the individuals and organisations involved[8].

In 2009, the heads of the Executive Council of the European Chiropractors' Union (ECU) and the European Academy of Chiropractic (EAC) involved in the European Committee for Standardization (CEN) process for the chiropractic profession set about establishing Chiropractic European Guidelines for Adverse Reactions (CEGAR) for a number of reasons: 1) to improve the overall quality of patient care, by aiming to reduce the application of potentially harmful interventions, and to facilitate the treatment of patients within the context of achieving maximum benefit with a minimum risk of harm; 2) to inform training objectives for the Graduate Education and Continuing Professional Development programmes of all 19 ECU member nations; and 3) to develop a guideline on patient safety standards since it is likely to become part of future CEN standards for ECU member nations.

The proposed guidelines herein are the result of a collaborative effort, involving not only members of a commission but also representatives of ECU member nations and chiropractic patients. They are based on a synthesis of the best available evidence and relevant published literature. The commission named the guidelines the European Guidelines for Chiropractic Incident Reporting and Learning Systems (EG-CIRLS) to emphasise the reporting and learning aspects. The main purpose of EG-CIRLS is to facilitate the introduction of an incident reporting culture within chiropractic practice in Europe, ultimately contributing to an improvement in the safety of patient care.

### Review process and phases of the development of the guidelines

In March 2009, a commission for the development of the guidelines was formed, consisting of seven members. All of the commission's members are European chiropractors working in six European countries, i.e. Belgium, Denmark, Norway, Spain, Switzerland and the United Kingdom. Four of the members work in private practice, and three work at chiropractic institutions. Most of the communication and work of the guidelines commission was done via online (closed-group) discussions in order to keep additional expenditure on resources, including travel to a minimum.

The commission decided to use the WHO Draft Guidelines for Adverse Event Reporting and Learning Systems[9] as a guide, and obtained permission to adapt a number of its chapters to the needs of the profession in Europe.

**Figure 1:**
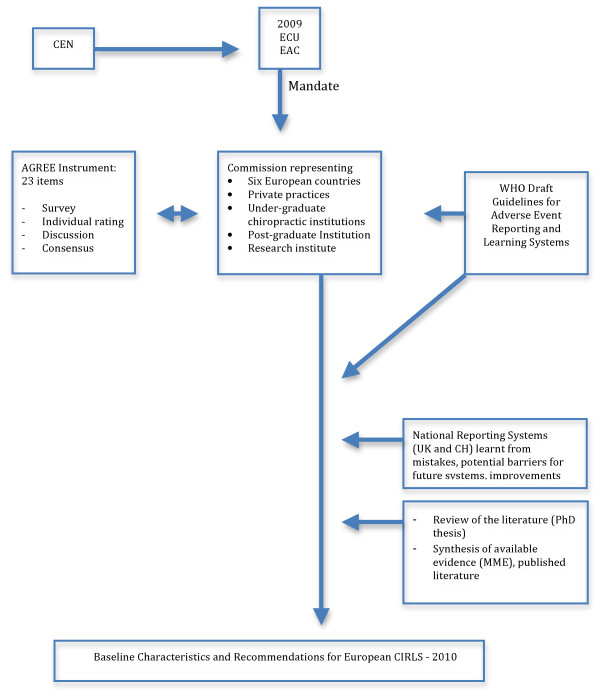
**Flow Diagram of Guidelines Developing Process**.

The "Appraisal of Guidelines for Research & Evaluation" (AGREE) instrument[10] was used in order to guide a structured and rigorous development methodology and to ensure that EG-CIRLS would meet minimum quality criteria. The AGREE instrument consists of 23 key items organised in six domains, with each domain intended to capture a separate dimension of guideline quality (Tables 1 and 2). The AGREE instrument was first used via an electronic survey sent to all seven members of the commission for its development process and quality (Table 1). Individual ratings are available in Additional file 1. The European Patient Organisation had rated the domain "Stakeholder involvement" in advance of the guidelines review (Additional file 2). The method used for formulating the recommendations was developed through consensus by all members of the commission at a meeting in Frankfurt in 2009. The group discussed the domain applicability, potential organisational barriers and costs in applying the recommendations, as well as review and control criteria and/or audit purposes. The final draft of the guidelines was again assessed by the commission members on the basis of all 23 AGREE items: first individually (n = 7), then by discussion and consensus (n = 5), and finally by individual ratings (n = 5) (Table 2). The overall development process for EG-CIRLS is summarised in Figure 1.

**Table 1:** Percentage of agreement with AGREE domains by the commission

Standardised Domain Score (EG-CIRLS)	Max possible scores	Min possible scores	Obtained scores	Percentage of agreement
Domain 1: Scope and purpose	84	21	78	90%

Domain 2: Stakeholder involvement	112	28	99	85%

Domain 3: Rigour of development	196	49	176	86%

Domain 4: Clarity and presentation	112	28	97	87%

Domain 5: Applicability	84	21	65	70%

Doman 6: Editorial independence	56	14	48	81%

The standardised domain score was calculated by summing up all the scores of individual items (4 point Likert scale) in a domain and by standardising the total as a percentage of the maximum possible score for that domain.

**Table 2:** Percentage of agreement with AGREE domains in regard to the final draft

Standardised Domain Score^I. ^(EG-CIRLS)	Max possible score	Min possible score	Obtained score	Percentage of agreement
Domain 1: Scope and purpose	60	15	45	67%

Domain 2: Stakeholder involvement	80	20	48	47%^II.^

Domain 3: Rigour of development	120	30	89	66%^III.^

Domain 4: Clarity and presentation	60	15	49	76%

Domain 5: Applicability	50	10	10	0%^IV.^

Domain 6: Editorial independence	40	10	40	100%

I. Was calculated by summing up all of the scores of the individual items (4 point Likert scale) in a domain and by standardising the total as a percentage of the maximum possible score for that domain; II. The domain "Stakeholder involvement" had been rated in advance of guidelines review by the European Patient Organisations (Additional file 2); III. Based on a previous literature review of a PhD thesis written at the University of Portsmouth. The method, used for formulating the recommendations, was developed through consensus by all members of the EG-CIRLS steering group at a meeting in Frankfurt in 2009; IV. The group discussed the domain Applicability. Potential organisational barriers and costs in applying the recommendations, as well as review and control criteria and/or audit purposes were considered. This item was judged as either being 'not applicable' or as 'strongly disagree'.

## Discussion

### Chiropractic and patient safety incidents

Providing health care of any kind, including the provision of chiropractic treatment, can be a complex and, at times, a risky activity. Safety in healthcare cannot be guaranteed, it can only be improved[11]. There will always be risks to patients, risks to practitioners and even risks to a profession as a whole. Capturing and recording information on patient safety incidents, and analysing this information, are essential steps to manage and reduce risk and, ultimately, improve patient safety.

So far, within Europe, information on patient safety incidents within the context of chiropractic treatment has been gathered in three ways: 1) data derived as a by-product of systems designed to investigate or respond to instances of poor quality care (for example, litigation for alleged medical negligence, a professional association's complaints procedure or cases referred to the national statutory regulator); 2) periodic internal or external studies and reviews[12,13] and 3) spontaneous reporting by individual chiropractors[14]. However, these sources of information can only give a haphazard and incomplete picture of the true nature and dimension of risks and adverse events associated with chiropractic treatment since they are usually based on individual cases, small studies in the population or rely on the review and interpretation of retrospective data often collected or initially reported by others. Furthermore, these methods are primarily reactive and strongly reflective of a blame culture, something that is not particularly conducive to individual learning or change of practice. Human error has traditionally been viewed as the factor that immediately precedes or precipitates an adverse event or serious failure. If something goes wrong at the "clinical sharp end" (that is, at the immediate practitioner-patient interface), it would seem obvious that an individual must have been responsible. However, we do know that humans are fallible and that errors are inevitable, even in the best-run organisations or systems. Errors must therefore be seen as consequences rather than causes, and as being shaped and provoked by "upstream" systemic factors. When an adverse event occurs, the crucial issue is not who caused the error but how and why the defences failed, and what factors contributed to create the conditions in which the error occurred[15]. We also know that there are often patterns and similarities in the sources of risk arising out of clinical practice, which would go undetected if incidents were not reported and analysed. Reporting is therefore the key to detect patient safety problems and can play a fundamental role in enhancing patient safety by subsequent learning from the failures and near misses that have occurred to others.

It is likely that terms such as "adverse event reporting" or "critical event audit" are likely to lead to apprehension and a feeling of intrusion amongst chiropractic clinicians and chiropractic organisations. It is therefore essential that a professional organisation encourages active learning from incidents in order to achieve an "informed culture" amongst its members. The airline industry provides evidence that "safety cultures", where open reporting and balanced analysis are encouraged in principle and by example, can have a positive and quantifiable impact on the performance of organisations[16]. Safety is therefore a strong feature of an informed culture, which has four critical components: a reporting culture; a just culture; a flexible culture; and a learning culture. These are presented in Table 3 (after Reason, 1997[16]).

**Table 3:** The critical components of an informed culture [15]

A **reporting culture**	creating an organisational climate in which individuals are prepared to report beneficial outcomes as well as adverse events or errors.
A **just culture**	not total absence of blame or disregard of individual responsibility, but an atmosphere of trust in which individuals are encouraged to provide safety related information.

A **flexible culture**	the skills and abilities of the individual are respected.

A **learning culture**	there is willingness and competence to draw the appropriate conclusions from its safety information systems, and the will to implement reforms where their need is indicated.

Another problem with classifying and identifying errors in healthcare is that there are so many ways of doing it. As outlined by Avery[17], one can focus on processes such as diagnosis or describe underlying systems failures. Alternatively, one can classify errors in terms of the type of disease, drug, or procedure most commonly associated with error, or in terms of the severity of outcomes. Risk and incident reporting systems are far less developed in primary care although far more patient contacts take place every year in a primary care setting. We know the therapeutic interventions and procedures are generally of a less serious nature, and nevertheless there is the potential for patients to be harmed by failures in care[15]. Almost all of the research conducted so far has concentrated on the epidemiology of medical error and its capture within hospital based secondary care largely because of a perception that this is where most serious incidents occur. The chiropractic profession, with its private primary care setting, therefore faces particular challenges in developing and maintaining an effective incident risk reporting system, not least because it is lacking some of the organisational structures, usually inherent within a national health care system, to support such systems.

Detecting and accurately recording incident errors are therefore fundamental steps in advancing clinical risk management by learning from experience. However, incident reporting should not solely focus on adverse outcomes. Not all unsafe actions, systems or situations will necessarily lead to bad outcomes all the time. The potential for an adverse outcome may exist, but for any number of reasons, for example timely detection or just sheer luck this outcome may not occur at all. This has been termed a "potential adverse event" or a "near miss"[15]. Near misses are therefore those incidents that do not result in harm and need to be clearly distinguished from adverse events and (Table 4). It is important for risk and safety management systems to collect and analyse information obtained from near miss situations since the knowledge gained as a result will proactively contribute towards the reduction of risk and prevention of harm.

**Table 4:** Types of events defined by WHO Guidelines [9]

Types of events	Definition The definitions of the three types of events are direct quotes of WHO Guidelines[9]
**Error**	Error has been defined as "the failure of a planned action to be completed as intended (i.e. error of execution) or the use of a wrong plan to achieve an aim (i.e., error of planning)". Although reporting of errors, whether or not there is an injury, is sometimes done within institutions, if reporting of all errors is requested the number may be overwhelming. Therefore, some sort of threshold is usually established-such as "serious" errors, or those with the potential for causing harm (also called "near misses" or "close calls"). Establishing such a threshold for a reporting system can be difficult. Hence, most "error reporting systems" are actually "adverse events caused by errors" systems.

**Adverse Event**	An adverse event is an injury related to medical management, in contrast to a complication of disease. Other terms that are sometimes used are "**mishaps**", "**unanticipated events**" or "**incidents**", and "**accidents**". Most authorities caution against use of the term accident since it implies that the event was unpreventable. Adverse events are not always caused by an error. For example, one form of adverse drug event, "adverse drug reaction" is, according to the WHO definition, a complication that occurs when the medication is used as directed and in the usual dosage. Adverse drug reactions are, therefore, adverse drug events that are not caused by errors. Many adverse events are caused by errors, either of commission or omission, and do, in fact, reflects deficiencies in the systems of care. Some reporting systems require that only preventable adverse events be reported, while others solicit reports whether or not a medical error occurred. One advantage of focusing reporting on adverse events rather than on errors is that it is usually obvious when a mishap has occurred; actual events focus attention. Comment by the authors of EG-CIRLS: *An adverse event is the result of a care delivery problem related to chiropractic management, in contrast to complications of disease. Chiropractic management includes all aspects of care, including diagnosis and treatment, failure to diagnose or treat, and the systems and equipment used to deliver care. Adverse events may be preventable or non-preventable. Preventable adverse event: An adverse event caused by an error or other type of systems or equipment failure*.

**Near miss** or **Close call**	A near miss" or "close call" is a serious error or mishap that has the potential to cause an adverse event, but fails to do so by chance or because it was intercepted. It is assumed (though not proven) that the underlying systems failures for near misses are the same as for actual adverse events. Therefore, understanding their causes should lead to systems design changes that will improve safety. A key advantage of a near miss reporting system is that because there has been no harm the reporter is not at risk of blame or litigation. On the contrary, he or she may be deserving of praise for having intercepted an error and prevented an injury. This positive aspect of reporting of near misses, has led some to recommend near miss systems for internal reporting systems within health-care organizations or other health-care facilities where a blaming culture persists. However, any hospital [*or private chiropractic practice*] that is serious about learning will also invite reports of near misses.

### Understanding the causes of adverse events

**Figure 2:**
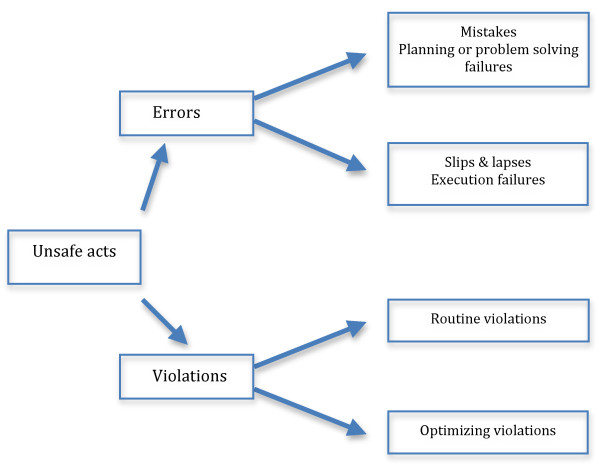
**Unsafe Acts: adapted from Reason, 1995**[20].

Reason[18] defines error as "the failure of planned actions to achieve their desired goal", and describes two ways in which this failure can occur (Figure 2). According to Reason[19], there are two approaches to the problem of human fallibility: the person and the system approaches. Each model gives rise to different attitudes to error management, and a basic appreciation of these differences is helpful to understand how clinical mishaps can occur. The person-centred approach focuses on the errors of individuals, blaming them for inattention, forgetfulness or carelessness. The person-centred approach is still the dominant model in medicine and healthcare at large. From an emotional perspective, the blaming of individuals is arguably more satisfying and easier than targeting institutions, organisations or systems[19]. This approach treats errors as moral issues, and as such isolates unsafe acts from their context, thus making it very difficult to uncover and eliminate recurrent error traps within a system[15]. The systems approach on the other hand, takes a more holistic stance on the issue of error and failure, concentrates on the conditions under which individuals function, and relates to the defences to avert errors or to minimise their effects. It acknowledges that humans are fallible and that errors are inevitable, even in the best-run organisations or systems. Errors are seen as consequences rather than causes, and as being shaped and provoked by "upstream" systemic factors[15]. The human factor cannot be changed. However, the conditions under which people work can be altered so as to make them less error provoking (please refer to Additional file 3 and 4 for further details)[[15,18-21])].

### Current national chiropractic reporting systems in Europe

Early efforts linked to the management of clinical risks within health care, including chiropractic, were primarily related to the setting up of processes in an attempt to control litigation and to reduce associated costs. Due to the increasing move in the late twentieth century towards documenting and learning from patient safety incidents, individuals within the chiropractic profession realised that it was paramount to become part of this developing safety culture. With this in mind, separate chiropractic patient incident reporting systems were developed in the United Kingdom and in Switzerland where chiropractors are committed to gathering all data relative to risks.

In 2005, the Anglo-European College of Chiropractic (AECC), in conjunction with the British Chiropractic Association (BCA), introduced the 'Chiropractic Reporting and Learning System' (CRLS) to collect patient safety incident data from BCA members[22]. It was taken up by the student clinics at AECC and, in modified form, at the Welsh Institute of Chiropractic, and subsequently rolled out to members of the Scottish Chiropractic Association. Although available to approximately 1600 of the UK's chiropractors, the initial take-up had been low[23]. Lack of awareness of the system and the types of incident that should be reported as well as fear and confusion regarding anonymity and the medico-legal implications of submitting reports were identified as key in explaining this underutilisation[22]. A second system known as the Patient Incident Reporting and Learning System (PIRLS) was developed at the McTimoney College of Chiropractic during 2007[24]. PIRLS was launched by the McTimoney Chiropractic Association ensuring incident reporting was available to a further 600 UK chiropractors. In order to unify the process of safety incident reporting in the UK and to facilitate participation among all 2500 UK chiropractors, the College of Chiropractors, the three UK chiropractic educational institutions, and the four UK professional associations combined their experiences in a joint project to develop a new online reporting system known as the Chiropractic Patient Incident Reporting and Learning System (CPiRLS).

The CPiRLS project aims to enhance the learning element and improve the ease and accessibility of incident reporting, to help educate chiropractors about the types of incidents they should report and to reassure chiropractors that the administration of incident reporting is independent, secure and anonymous such that they have nothing to fear by sharing their experiences. The project forms part of a wider initiative to further enhance the culture of safety within the UK chiropractic profession. The CPiRLS website[http://www.cpirls.org] informs all visitors of the purpose and nature of incident reporting and learning but, in its initial form, is set up such that only chiropractors can submit and read reports. This is ensured through secure access with a universal password available only to chiropractors via the membership areas of their association websites. The universal nature of the password and design of the website database mean that individuals submitting reports cannot be identified by anybody, including those administering the system. This was felt to be essential if chiropractors are to feel comfortable and secure in submitting and sharing reports without fear of legal retribution. The CPiRLS online reporting form is provided in three versions according to whether the incident under report has either happened, nearly happened (near miss) or has been identified as an incident waiting to happen (following identification of an error or discrepancy of process for example).

Users start by choosing between these three types of incident and then progress through the form explaining what happened, why it happened and what actions were taken. Drop-down lists and radio buttons assist simple and rapid completion of the form. Submitted reports are published in outline form on the website. Users who are logged in to the site can read these reports and submit comments. This sharing of information and interaction among peers is designed to maximise the learning aspect of CPiRLS. All submitted material is monitored by CPiRLS team members who can edit inappropriate matter and access/download all data for future thematic analysis. The CPiRLS initiative is actively addressing the current underutilisation of incident reporting as a learning tool and will lead to the regular publication, by the CPiRLS team, of alerts and detailed guidance to assist chiropractors in managing risk more effectively.

It is primarily legislation (Swiss Sickness and Accident Insurance and the Swiss

Law on Medical Professions) that drives quality management for patient safety in chiropractic practice in Switzerland. The increasing awareness and political commitment to improve safety affects all health care sectors including the chiropractic profession. The need for health professionals to continually improve quality and enhance patient safety is omnipresent. Unfortunately, the majority of well developed critical incident reporting systems are implemented in clinical inpatient and hospital settings, almost none of them in private medical or chiropractic practices. These facts and the low reporting rate cited in the UK study conducted by Thiel and Bolton[22] encouraged the Swiss Chiropractic Association to further investigate chiropractic incident reporting, its promotion and implementation. A first reporting and learning project, the Swiss Critical Reporting and Learning System (CRLS) was launched in September 2007 by Wangler and Zaugg[25].

Regular patient safety training is not yet established in chiropractic. In order to promote a change in attitudes towards greater patient safety, information and education should be part of the training of future chiropractors. With the help of a literature synthesis[26], 10 factors developed by Bland and co-workers[27] were adapted for successful promotion of patient safety competence in private practice, i.e. reporting and learning from adverse events in chiropractic care. The annual National Continuing Education Convention (2007) was considered to be the ideal environment to introduce and promote this first reporting and learning project. A survey on chiropractors' readiness and capacity for patient safety attitude change using the

Safety Attitude Questionnaire (SAQ)[28,29] for ambulatory care was conducted to assess the competencies of Swiss chiropractors in relation to patient safety issues.

The project consisted of a number of instructional approaches, such as providing written documentation, holding platform presentations and facilitating large and small group discussions incorporating feedback by experts.

Qualitative analysis[25] indicated that the biggest challenge seemed to be the culture shift from blame to trust. Lecturing is convenient, but it did not change behaviour in practice. As with clinical reasoning, reporting cannot occur in a vacuum, but must be built into daily practice. Reporting and learning skills have first to be developed and practised. An online discussion forum between experts and practitioners could facilitate the development of such skills. Active support by leaders of the national association was minimal. Finally, safety and quality have to be integrated into continuing professional development.

The Swiss CRLS website http://www.crls-chiro.ch informs on the purpose and scope of incident reporting and learning. As with the UK CPiRLS, only chiropractors can submit and read reports on the password-protected website. Reports and discussions are kept totally anonymous. The password-secured forum is user-friendly and the reporting procedure is clear. Different to the CPiRLS system in the UK, the Swiss chiropractor describes the incident with first reflection and without categorisation. This means the user simply analyses what went wrong and what action should be taken. Regular, timely and effective feedback by experts regarding this proposed action is essential.

Approximately six months after the start of the reporting and learning project the frequency with which reports were submitted diminished significantly. Therefore another pilot project, dealing with structured and systematic analysis of real-life adverse events in chiropractic care, was initiated[30]. The London Protocol[31] was chosen as the analysis method.

### WHO Draft Guidelines for Adverse Event Reporting and Learning Systems

Of utmost importance for a reporting system is the appropriate use of the information it produces. Reporting is a tool for obtaining safety information and to advance quality improvement and error prevention. According to the WHO Draft Guidelines for Adverse Event Reporting and Learning Systems [9], successful patient safety reporting systems have the following characteristics: (1) Reporting must be safe for the individuals who report; (2) reporting leads to a constructive response; (3) reporting is only of value if it leads to meaningful analysis; and (4) learning requires expertise and adequate financial resources i.e. the agency that receives reports must be capable of disseminating information and making recommendations for changes, and informing the development of solutions.

The WHO has produced Draft Guidelines for Adverse Event Reporting and Learning Systems[9] that are derived from experiences both in healthcare and in other industries, particularly aviation. Table 5 lists the characteristics and Table 6 the requirements that have been identified by various authors as essential to the success of any reporting systems concerned with patient safety in these guidelines.

**Table 5:** Characteristics of Successful Reporting Systems[9]*

Non-punitive	Reporters are free from fear of retaliation against themselves or punishment of others as a result of reporting.
**Confidential**	The identities of the patient, reporter, and institution are never revealed.

**Independent**	The reporting system is independent of any authority with power to punish the reporter or the organization.

**Expert analysis**	Reports are evaluated by experts who understand the clinical circumstances and are trained to recognize underlying system causes

**Timely**	Reports are analyzed promptly and recommendations are rapidly disseminated to those who need to know especially when serious hazards are identified.

**System-oriented**	Recommendations focus on changes in systems, processes, or products, rather than being targeted at individual performance.

**Responsive**	The agency that receives reports is capable of disseminating recommendations. Participating organizations commit to implementing recommendations whenever possible.

*Reproduced with permission of WHO Press http://www.who.int/about/licensing/en

**Table 6:** Requirements of Successful Reporting Systems [9]*

Key Messages Capacity to respond to reports	
Clear objectives	Capacity to respond to reports
Clarity about who should report	A method for classifying and making sense of reported events
Clarity about what gets reported	The capacity to disseminate findings
Mechanisms for receiving reports and managing data	Technical infrastructure and data security
Expertise for analysis	

*Reproduced with permission of WHO Press http://www.who.int/about/licensing/en

### Characteristics and Recommendations for European CIRLS

A reporting system needs to have a clear purpose and clear objectives. It must be obvious to the user who should report and what must be reported. It must allow users to understand the purpose of reporting and assure them that there will not be any retaliation or punitive measures taken as a result of their reporting. The mechanisms for receiving reports and managing the data must guarantee protection for the patient, the reporter and any institution involved. Once the data have been obtained, experts must be available to analyse the specific situation, circumstances and any contributing factors related to the reported incidents. All facts need to be accumulated in an integrated fashion, thus generating manageable information. Timing is of utmost importance so that the information resulting from this process is distributed efficiently through the right channels and amongst those parties who need to access it within an appropriate time span. This measure means that serious hazards, once identified, are prioritised quickly. Information needs to be converted into recommendations that will promote changes that go beyond individual performance, and target systems and operational structures. Agencies receiving the reports need to develop a technical infrastructure that will allow them to receive data, process the information and generate recommendations for the purpose of providing a learning experience for the reporter and organisations alike. This will achieve an improved safety environment for patients, where prevention is one of the cornerstones.

The commission members deliberated on the characteristics and recommendations for European Chiropractic Incident Reporting and Learning Systems and arrived at a number of criteria and requirements for successful reporting of, and learning from incidents. These are outlined in Table 7. Most recommendations were derived from the WHO draft guidelines[9].

**Table 7:** Recommendations for European Chiropractic Incident Reporting and Learning Systems, developed by the commission (2010)

Criteria for success	Requirements for success
Clear purpose	Strategy to communicate the purpose of learning to improve patient safety in practice

Clear strategy	Specific instructions for reporters (e.g., chiropractors and staff) on incidents to be reported

Anonymity	Safe IT-systems and procedures to guarantee anonymity/confidentiality for patients, practitioners and institutions

Risk free	Administrators and experts involved in managing the system without authority for punishment and retaliation

Feedback to the reporting individual	Experts must analyse the incidents and give timely feedback. Focus should be on improving the clinical setting and/or avoiding similar incidents in the future

Feedback to organizations	If serious hazards are identified, information - after being made anonymous - should be distributed to organisations and/or the individual through pre-determined channels without delay

Accumulation of knowledge	Received data should be collated in a structured fashion to allow meaningful analyses

Formulation of guidelines	An expert panel should be appointed to transform the aggregated analyses into clinically meaningful guidelines, targeting systems and operational procedures as well as individual performance

Implementation	A comprehensive strategy for national implementation must be in place for the individual countries, including (but not limited to) presentation at national meetings, workshops and making available written information for clinicians

## Conclusions

Before a reporting and learning system can be established it has to be clear what the objectives of the system are, what resources will be required and whether the organisation responsible for implementation has the capacity to respond to reports. This requires the availability of experts to analyse the incidents and to provide feedback in a timely fashion. A comprehensive strategy for national implementation must be in place including, but not limited to, presentations at national meetings, written information to all practitioners and workshops to ensure standardised reporting. Training and support must be available for both experts and reporting clinicians. Patterns and similarities in the sources of risk arising out of clinical practice must be disseminated to individual practitioners and appropriate organisations and institutions. Anonymity and confidentiality for patients and practitioners must be guaranteed at any time.

When implementing a national CIRLS in Europe, special consideration must be given to cultural differences, legal obligations, available human and financial resources, as well as the commitment of an organisation's leadership to such a project as evidenced by the two existing systems in the UK and in Switzerland.

### Considerations for the Future

A number of European patient safety incident reporting systems offer the possibility for patients or members of the public to report directly, either via a separate pathway designed for public and patient reporting (e.g., http://www.nrls.npsa.nhs.uk/report-a-patient-safety-incident) or by allowing anyone to access and utilise the same reporting format (e.g., http://www.jeder-fehler-zaehlt.de). We foresee a future mechanism whereby chiropractic patients could report incidents directly and independently. However, in order to achieve a change of culture within the profession (feel secure to report), a password-protected system, restricted to chiropractors only, is likely to be the immediate way forward.

## Competing interests

The authors declare that they have no competing interests.

## Authors' contributions

MW was responsible for: the methodology used to collect and to summarise the evidence, leading the online discussions, organising and participating in the analysis of the collected data, formulating recommendations and conclusions, and drafting and finalising the manuscript.

HT and BZ were responsible for: the literature review, the methodology used to collect and to summarise the evidence, participating in the analysis of the collected data, formulating recommendations and conclusions, and reviewing and finalising the manuscript.

TM, RF, LH, and TJR were responsible for: summarising the evidence, participating in the analysis of the collected data, formulating recommendations and conclusions, and reviewing the manuscript.

All authors have read and approved the final abstract and manuscript.

## Supplementary Material

Additional file 1 **Percentage of agreement with the AGREE domains by members of the commission (n = 5)**. Click here for file

Additional file 2 **The European Patient Organizations' review**. Click here for file

Additional file 3 **Summary on person and system approach**. Click here for file

Additional file 4 **The "Swiss Cheese Model" of accident causation [16]**. Click here for file
