# Supplementary material for: Creating European guidelines for Chiropractic Incident Reporting and Learning Systems (CIRLS): relevance and structure
Source: Chiropr Man Therap. 2011 Apr 1;19:9. doi: 10.1186/2045-709X-19-9 (PMC3079683; doi:10.1186/2045-709X-19-9)
Supplement: Additional file 1 — Percentage of agreement with the AGREE domains by members of the commission (n = 5). [file 2045-709X-19-9-S1.DOC]

**Additional file 1 - Percentage of agreement with the AGREE domains by members of the commission (n = 5)**

| Reviewers | ITEMS | | | | | | | | | | | |
| --- | --- | --- | --- | --- | --- | --- | --- | --- | --- | --- | --- | --- |
|  | 1 | 2 | 3 | 4 | 5 | 6 | 7 | 8 | 9 | 10 | 11^IV^ | 12 |
| A | 4 | 1 | 4 | 2 | 1^I^ | 4 | 2 | 3^II^ | 3 | 3^III^ | NA | 3 |
| B | 4 | 1 | 4 | 3 | 1 | 4 | 2 | 3 | 3 | 3 | NA | 3 |
| C | 4 | 1 | 4 | 2 | 1 | 4 | 3 | 2 | 2 | 3 | NA | 3 |
| D | 4 | 1 | 4 | 2 | 1 | 4 | 2 | 3 | 3 | 3 | NA | 3 |
| E | 4 | 1 | 4 | 2 | 1 | 4 | 3 | 2 | 2 | 2 | NA | 3 |
| **TOTAL** | **20** | **5** | **20** | **11** | **5** | **20** | **12** | **13** | **13** | **14** | **-** | **15** |
| Reviewers | ITEMS | | | | | | | | | | | |
|  | 13 | 14 | 15 | 16^VI^ | 17 | 18 | 19^V^ | 20 | 21 | 22 | 23 |  |
| A | 4 | 2 | 4 | NA | 4 | 2 | NA | 1 | 1 | 4 | 4 |
| B | 4 | 3 | 4 | NA | 4 | 2 | NA | 1 | 1 | 4 | 4 |
| C | 4 | 3 | 4 | NA | 4 | 2 | NA | 1 | 1 | 4 | 4 |
| D | 4 | 3 | 4 | NA | 4 | 2 | NA | 1 | 1 | 4 | 4 |
| E | 4 | 3 | 3 | NA | 4 | 2 | NA | 1 | 1 | 4 | 4 |
| **TOTAL** | **20** | **14** | **19** | **-** | **20** | **10** | **-** | **5** | **5** | **20** | **20** |

This rating was done in London in May 2010 by 5 of 7 members of the commission.

I Only the ProChiropractic Europe has been addressed (Additional file 2). The group decided that patients’ view and preferences must be sought in future too; II Based on a previous literature review of PhD thesis written at the University of Portsmouth; III The method, used for formulating the recommendations, was developed through consensus by all members of the commission at a meeting in Frankfurt in 2009; IV The external peer review will be done at publication in a peer reviewed journal; V Item 19 (Potential organizational barriers in applying the recommendations has been referenced in the text but not discussed in detail by these guidelines; VI Item 16 (Different options for management of the condition) cannot be applied to these guidelines.
