# Supplementary material for: Creating European guidelines for Chiropractic Incident Reporting and Learning Systems (CIRLS): relevance and structure
Source: Chiropr Man Therap. 2011 Apr 1;19:9. doi: 10.1186/2045-709X-19-9 (PMC3079683; doi:10.1186/2045-709X-19-9)
Supplement: Additional file 2 — The European Patient Organizations' review. [file 2045-709X-19-9-S2.DOC]

**Additional file 2 – The European Patient Organizations’ review**

| Dear Dr Wangler  RE: European Guidelines for Chiropractic Incident Reporting &Learning Systems (EG-CIRLS) Thank you for asking ProChiropractic Europe to provide you with a view on the final draft of the European Guidelines for Chiropractic Incident Reporting & Learning Systems (EG-CIRLS). It has been very interesting to read your “work in progress” and I congratulate you and your team on the depth and breadth you have achieved with this document.  The World Health Organisation clearly supports the importance of guidelines in relation to patient safety, and as a patient organisation we can only “feel very safe” to be involved at this early stage of developments. I can foresee that an incident reporting and learning system at a national, or perhaps even European level, would be a wonderful institution for chiropractors, who had encountered patient related incidents in their practice, to turn to for help and guidance. I think that it is a great idea that only chiropractors can submit and read reports via password secured access. This is the only way chiropractors will feel secure when submitting a report. But what about the patient? Will s/he also be able to turn to this institution or a reporting system to either report her/his experiences or to seek support? If, in the future, there is to be a way for chiropractic patients to be able to report themselves, it will be important that any information or documentation related to such a patient- based system will be provided in a format which will be easy to read and understand by lay people. It could also be an opportunity for chiropractic patient organisations to become involved and perhaps work together with national or pan- European reporting and learning systems.  I would like to thank you and the board of EG-CIRLS for their interest in ProChiropractic Europe and approaching me for my input. I look forward to the outcome of the survey. Yours sincerely  Ann-Liss Taarup, President ProChiropractic European; www.ProChiropactic.org; June 26 2010 |
| --- |
