# Supplementary material for: Creating European guidelines for Chiropractic Incident Reporting and Learning Systems (CIRLS): relevance and structure
Source: Chiropr Man Therap. 2011 Apr 1;19:9. doi: 10.1186/2045-709X-19-9 (PMC3079683; doi:10.1186/2045-709X-19-9)
Supplement: Additional file 3 — Summary on person and system approach. [file 2045-709X-19-9-S3.DOC]

**Additional file 3 – Summary on person and system approach**

| Person Approach | Focus on the psychological precursors to error and based on the aetiology of unsafe acts such things as slips, lapses and mistakes, and violations [15]. Slips and lapses are failures in the execution of an adequate plan, whereby slips relate to observable actions and are associated with failures of attention. Lapses are more internal events and relate to failures of memory [18]. They usually occur during largely automatic performance of routine tasks in familiar surroundings.  Mistakes are failures of intention, where actions may go entirely as planned, but the plan is inadequate for achieving the intended outcome[18,21]. Violations can be necessary or situational, in instances where existing rules or procedures are deemed to be inappropriate in order to get the job done[18]. In contrast to errors, violations are usually deliberate deviations from safe operating practices, procedures, standards or rules. |
| --- | --- |
| System Approach | The idea central to the system approach is that of system defences. When an adverse event occurs, the crucial issue is not who caused the error but how and why the defences failed, and what factors helped to create the conditions in which the error occurred[15]. High risk industries, such as commercial aviation and the oil and the gas industry, have achieved exemplary safety performance by developing effective defences, barriers and safeguards that become interposed between the source of the hazard and the potential victim(s)[21]. As a result, these high technology systems have developed a series of defensive layers, and in an ideal world each of these layers would be completely intact. However, the human element can weaken or create gaps in these defences by either “active failures” or “latent failures or conditions”[21]. Active failures are the unsafe acts (errors and violations) committed by those at the sharp end of the system, i.e. in healthcare, people who are in direct contact with the patient and have an immediate and usually short-lived impact on the defensive layers. Latent failures or conditions, on the other hand, are very much comparable to resident pathogens in the body. By themselves, they often do no particular harm and may lie dormant in the system for long periods of time. It is only when they combine with active failures that the defensive layers are penetrated or bypassed [15,19]. Unlike active failures, whose precise forms are difficult to predict, latent conditions are always present, and as such, can be identified and removed. The “Swiss Cheese Model” is best demonstrated by the relationship between active and latent failures or conditions, and how gaps through several layers of defences within a system can arise. (Additional file 4: reproduced from UK Department of Health, 2000[15] with permission of Her Majesty’s Stationery Office). |
