# Supplementary figures and images for: Creating European guidelines for Chiropractic Incident Reporting and Learning Systems (CIRLS): relevance and structure
Source: Chiropr Man Therap. 2011 Apr 1;19:9. doi: 10.1186/2045-709X-19-9 (PMC3079683; doi:10.1186/2045-709X-19-9)

**Additional file 4 - The “Swiss Cheese Model” of accident causation [15]**

**
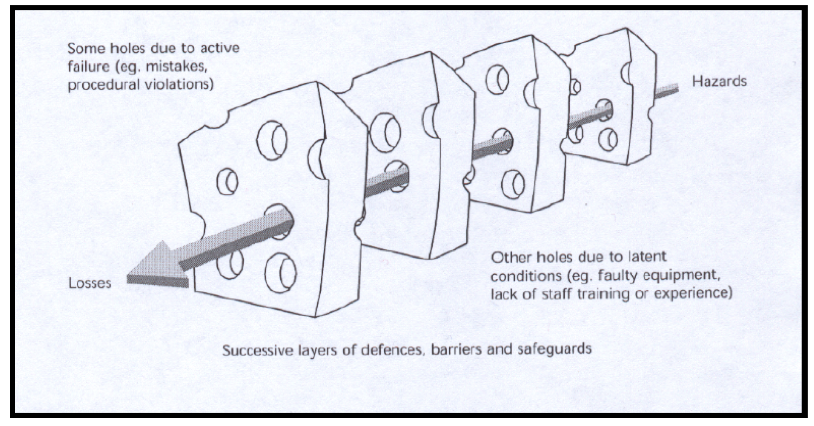
**

Supplement: Additional file 4 — The "Swiss Cheese Model" of accident causation [16]. [file 2045-709X-19-9-S4.DOC]
